# Cost effectiveness of intraoperative pathology in the management of indeterminate thyroid nodules

**DOI:** 10.20945/2359-3997000000263

**Published:** 2020-06-19

**Authors:** Christopher D. Vuong, WayAnne B. Watson, Daniel I. Kwon, Sonia S. Mohan, Mia N. Perez, Steve C. Lee, Alfred A. Simental

**Affiliations:** 1 Department of Otolaryngology- Head and Neck Surgery Loma Linda University Medical Center Loma Linda California United States Department of Otolaryngology- Head and Neck Surgery, Loma Linda University Medical Center, Loma Linda, California, United States; 2 Loma Linda School of Medicine Loma Linda University Medical Center Loma Linda California United States Loma Linda School of Medicine, Loma Linda University Medical Center, Loma Linda,California, United States; 3 Department of Otolaryngology-Head and Neck Surgery University of Southern California Los Angeles California United States Department of Otolaryngology-Head and Neck Surgery, University of Southern California, Los Angeles, California, United States; 4 Department of Pathology Laboratory Medicine Loma Linda University Medical Center Loma Linda California United States Department of Pathology and Laboratory Medicine, Loma Linda University Medical Center Loma Linda, California, United States

**Keywords:** Cost effectiveness, thyroid nodules, frozen section, thyroidectomy

## Abstract

**Objective:**

This study aims to determine the cost effectiveness of rapid frozen section (RFS) for indeterminate thyroid nodules.

**Materials and methods:**

A retrospective chart review was conducted between January 2009 and June 2013 at a tertiary care institution. Main outcomes were number needed to treat, RFS efficacy, and cost-savings of avoiding second completion thyroidectomy. Cost-effectiveness was estimated using 2015 Medicare reimbursement rate.

**Results:**

Out of 1,114 patients undergoing thyroid surgery, 314 had preoperative AUS/FLUS cytopathology and subsequent thyroid lobectomy with RFS. RFS identified 13 of the 32 patients with malignancy resulting in a total thyroidectomy. 19 of the 29 malignancies not detected by RFS were papillary microcarcinomas.

**Conclusions:**

Completion thyroidectomy was avoided in 1 out of every 24 patients resulting in cost-savings of $ 80.04 per patient. In the era of outpatient thyroid surgery, intraoperative RFS for indeterminate thyroid nodules is cost-effective.

## INTRODUCTION

Fine needle aspiration (FNA) is a fundamental and inexpensive method of evaluating thyroid nodules. Since its integration, FNA has succeeded in greatly reducing unnecessary thyroid surgery worldwide and continues to aid in the management of thyroid lesions ( 1 ). In 2007, the Bethesda System for Reporting Thyroid Cytopathology (BSRTC) was established as a means to stratify malignancy risk of FNA samples ( 2 ). The resulting recommendation was a six-tier classification system that predicted malignancy of nodules based on available literature: 0-3% for benign; 5%-15% for atypia of undetermined significance or follicular lesion of undetermined significance (AUS/FLUS), 15%-30% for follicular neoplasm or suspicious for a follicular neoplasm, 60%-75% for suspicious for malignancy, and 97%-99% for malignancy ( 2 , 3 ).

While the management of benign, malignant, or suspicious for malignancy FNA is generally straightforward, the “indeterminate” classes of AUS/FLUS and follicular neoplasm/suspicious for follicular neoplasm have proven to be more challenging ( 4 , 5 ). By definition, indeterminate thyroid nodules are specimens with architectural and/or nuclear atypia that cannot confidently be classified as benign or malignant, therefore present more challenges when making therapeutic recommendations. Currently, the recommended management for cytopathology of AUS/FLUS is to repeat FNAB in 3 to 6 months in the absence of other factors favoring surgery such as ultrasound findings of greater than 2cm, radiation exposure, family history, age greater than 40, male sex or patient preference ( 5 , 6 ). Patients with persistent indeterminate cytology may receive diagnostic thyroid surgery ( 5 ).

The added value of rapid frozen section (RFS) during diagnostic thyroid lobectomy is generally undetermined. As complete thyroid ablation is often preferred by both patient and clinician in the case of malignancy, rapid frozen sections may aid in the intraoperative decision to continue with total thyroidectomy by detecting malignancy in patients with indeterminate cytology. RFS is of limited usefulness in detecting follicular carcinoma ( 5 , 7 - 9 ) but can be more useful in the setting of papillary thyroid carcinoma. Some shortcomings of RFS include sampling error, potential wasting of diagnostic material in low volume lesions, and is dependent upon the pathologic expertise and experience ( 10 ). However, the alternative is the re-operation in the form of completion thyroidectomy after final pathologic analysis is completed which introduces significant burden to the patient, surgeon and healthcare system. Thus, a cost-effectiveness analysis is a useful tool in order to determine whether the additional expense of intraoperative RFS results in the improved outcome of overall cost savings when included in the diagnosis of patients with indeterminant thyroid nodules. This study analyzes the cost-effectiveness of routine intraoperative consultations in patients with AUS/FLUS diagnosis on fine needle aspiration.

## MATERIALS AND METHODS

We obtained approval from our institutional review board for a study protocol to conduct a retrospective chart review at our center between January 2007 and June 2013, identifying 1114 consecutive patients who underwent primary thyroid surgery for suspected neoplasm based on pre-operative fine-needle aspiration (FNA). Of these patients, 314 were identified who had a preoperative FNA diagnosis of Bethesda III (AUS and FLUS). These patients underwent thyroid lobectomy with intraoperative decision to proceed to total thyroidectomy if RFS indicated malignancy. Patients who had thyroid surgery for indications other than suspected neoplasm and patients who had initial thyroid surgery elsewhere were excluded, regardless of their final pathology. Only patients who had preoperative FNA, intraoperative RFS, and final pathology reports were included in the final study cohort.

Patients had preoperative counseling on risks and benefits of thyroid lobectomy versus total thyroidectomy. They were consented for thyroid lobectomy with possible total thyroidectomy based on the frozen section diagnosis.

FNA, RFS and final pathology data were collected on all patients. FNA diagnoses were categorized on all patients using the Bethesda system (BSRTC). FNA cytopathologic samples from outside our institution were reviewed and categorized internally. RFS diagnoses were categorized as benign, malignant, or indeterminant to be deferred to final pathology.

Correlation between RFS and final pathology was done calculating sensitivity, specificity and diagnostic accuracy. Costs were estimated from Medicare-based reimbursement rates for physician-provided services (surgeon, anesthesia and pathology fee schedules) and facility costs to determine the relative value of routine intraoperative pathology. Thyroid surgery is routinely done on an outpatient basis at our institution, and therefore facility costs were estimated using Hospital Outpatient Prospective Payment (HOPP) system developed by Centers for Medicare & Medicaid Services (CMS) ( Table 1 ). Healthcare Common Procedure Coding System (HCPCS) codes were for partial removal of thyroid, total thyroidectomy, and repeat thyroidectomy were 60220, 60240, 60260, respectively, and Ambulatory Payment Classification (APC) codes were 0114, 0114, 0256.

**Table 1:** Medicare Reimbursement Rates for Hospital Services (USD$)

	Thyroid lobectomy	Total thyroidectomy	Complete thyroidectomy
Hospital Outpatient Facility Fees	4,240	4,240	3,730
Anesthesia (60 minutes)	211	211	211
Additional Anesthesia (30 minutes)		43	
Surgeon Fee	735	952	885
Frozen Section Consultation	109	109	
Totals	5,295	5,555	4,826

## RESULTS

**Figure 1:**
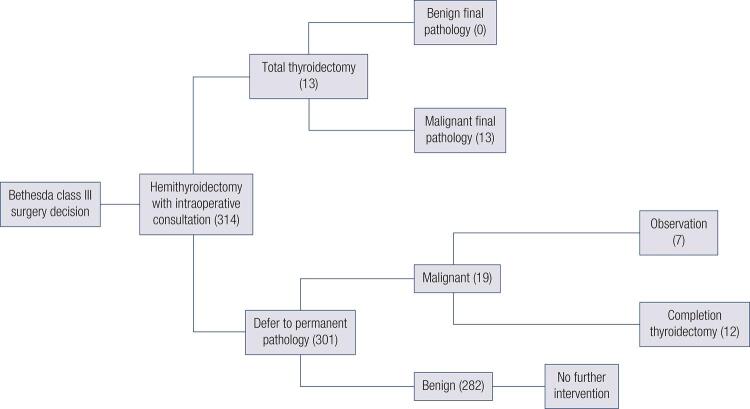
Results of Bethesda class III surgery with intraoperative consultation at our institution.

Of the 1114 patients initially identified who had thyroid surgery for neoplasia, 314 patients had a preoperative FNA showing AUS or FLUS and underwent intraoperative RFS. Of the 314 AUS/FLUS cases, 32 were diagnosed with malignancy on final pathology ( Table 2 ). Intraoperative RFS was able to detect malignancy in 13 of the 32 patients resulting in changing the surgery from hemithyroidectomy to total thyroidectomy during the same surgery. RFS failed to detect malignancy in 19 patients who eventually had malignancy seen on final pathology. All RFS diagnoses of malignancy were confirmed by final pathology without any false positives. Of the false negatives, 7 cases had micro-papillary carcinoma. These patients elected for observation and did not have additional surgery. However the remaining 12 patients with final pathologic diagnosis of malignancy missed by RFS chose to undergo completion thyroidectomy ( Figure 1 ).

**Table 2:** RFS pathology correlation to final pathology

RFS pathology	Final pathology
Benign 280	Benign 267 Conventional papillary 8 Papillary microcarcinoma 5
Malignant 13	Conventional papillary 13
Indeterminant 21	Benign 15 Conventional papillary 4 Papillary microcarcinoma 2

In 314 patients with preoperative FNA showing AUS or FLUS, RFS was able to detect malignancy in 13 patients intraoperatively guiding the surgical decision for total thyroidectomy. In our surgical series, for every 24 patients with preoperative FNA showing AUS or FLUS, RFS allowed one patient to avoid a second surgery ( Figure 1 ).

The additional cost of intraoperative RFS analysis ($ 109) for each patient was compared with the cost of avoiding completion thyroidectomy ($ 4826) in one out of every 24 cases of preoperative AUS/FLUS. The routine use of RFS in AUS or FLUS at our institution resulted in approximate savings of $ 80.04 per surgery in this population ( Table 3 ).

**Table 3:** Cost savings of performing a Total versus Completion thyroidectomy in cases where malignancy was confirmed by rapid frozen section (USD$)

	Number of cases	Cost per case	Total cost
Completion Thyroidectomies Avoided	13	4,826	62,738
Added Cost of Total Thyroidectomy	13	260	-3,380
Frozen Section Costs	314	109	-34,226
Total Net Savings			25,132
Savings per Case			80.04

## DISCUSSION

Fine-needle aspiration cytopathology has excellent utility in the evaluation of thyroid nodules and the Bethesda System for Reporting Thyroid Cytopathology helps guide therapeutic recommendations of these nodules according to their malignant potential. Of the patients who undergo fine needle aspiration, approximately 30% are classified as indeterminate (Bethesda III and IV) ( 11 , 12 ). For equivocal cytopathology, treatment recommendations are less straightforward and involve an informed discussion between patient and physician regarding observation, surgery, and extent of surgery.

Rapid frozen section may be a useful tool in guiding management during diagnostic lobectomy and avoid the need for a secondary operation. Second surgery may result in additional anesthetic risks, possible costs from copays, travel, and lost productivity for the additional time off work for family and patient. Intraoperative consultation for thyroid nodules have declined since the implementation of FNA due to the accuracy of FNA in diagnosing papillary thyroid carcinoma preoperatively and need for extensive sampling to identify follicular carcinoma intraoperatively ( 7 - 9 , 13 - 15 ). The patients in our series elected for surgical management of their AUS/FLUS lesion after appropriate workup counseling, including discussion on the potential for thyroid lobectomy alone in selected low-risk papillary thyroid carcinomas, and subsequently endorsed their desire for total thyroidectomy in the case of malignancy detected on RFS. In 10% of cases, AUS/FLUS-diagnosed lesions were found to harbor malignancy on final pathology which is consistent with other studies ( 2 ). With a RFS specificity of 100%, our results are consistent with other studies which also report a specificity of 99% to 100% ( 4 ,5,7-9,16). This demonstrates that a RFS diagnosis of malignancy is highly reliable. In contrast, RFS had a sensitivity of 40% and failed to detect malignancy in 19 cases which is also mirrored by other series which range from 29%-55% in follicular carcinoma and to 68%-77% of papillary carcinoma ( 5 , 7 - 9 ). Reasons for false negatives include the inability to detect invasion in follicular carcinoma and inaccurate sampling for small foci of malignancy such as papillary microcarcinoma. After omitting the patients with papillary microcarcinoma, the routine usage of RFS was able to detect malignancy and avoid potential second surgery in 13 of 312 patients. It is also possible that routine use of touch prep cytology may have increased the malignancy detection rate but use of touch prep is pathologist dependent in our institution and is not recorded. Therefore, the contribution of touch prep to the diagnostic yield or cost efficiency of RFS is not addressed by this study.

Routine frozen section at our institution in patients receiving surgery for AUS/FLUS lesions resulted in net savings of $ 80.04 per patient. Estimated cost of surgery was based on CMS reimbursement rates for surgeon, anesthesia, and facility fee contrasted against the additional cost of anesthesia time for total thyroidectomy and intraoperative RFS. Time-dependent anesthesia fees were estimated using average operative times at our institution. Conversion to total thyroidectomy after positive RFS was estimated to be an additional 30 minutes. Average time to perform RFS at our institution is 17 minutes, which was negligible in terms of additional operative time as diagnosis is usually obtained before the surgical wound is closed.

Usage of RFS has shown to be cost saving in some other studies. Roach and cols. ( 4 ) showed a net savings of $ 1298 per patient based on conversion of 17% of the diagnostic lobectomies to total thyroidectomy. In another study, Lai and cols. study of 169 patients with indeterminate classification, frozen section was able to identify malignancy in 30.1% of cases yielding cost savings of $ 22.01 per patient in the Canadian health care system ( 17 ). On the other hand, McHenry and cols. and Chen and cols. deduced that routine frozen section did not change management and was not cost-effective ( 18 , 19 ). The differences in costs of each study may be due to the heterogeneity of study design. For example, some studies calculated hospital charges instead of Medicare reimbursement while other reports were based on inpatient costs instead of outpatient thyroidectomy which have become increasingly the standard of practice. In addition, our study focuses on Bethesda class III nodules rather than both classes III and IV. To our knowledge, this is the largest series examining the utility of RFS in the context of class III nodules.

Repeat surgery carries additional costs and burdens that are more difficult to calculate. Each additional surgery confers additional mortality risk, wound complications, as well as the specific risks of thyroid surgery. From the patient perspective, each surgical event also poses social burdens such as time off work, time away from family, recovery period, disruption of normal schedules, and other intangibles such as pain and anxiety. Hamburger and Hamburger ( 20 ) deduced that most patients would actually prefer a definitive operation for RFS-diagnosed cancer rather than undergo a secondary procedure. Additional surgery also creates a burden on the healthcare system in terms of time and resources that could be utilized with another patient.

In our study, RFS was used only in patients who desired complete removal of thyroid in case of malignancy. Thus this study assumed that intraoperative decision for total thyroidectomy would not increase risks such as hypoparathyroidism, nerve injury or the cost a thyroid hormone replacement as these patients would likely have undergone completion thyroidectomy regardless.

Limitations to this study include the easy investigation of estimation of cost and the possible inadequacy of our chosen methodology in terms of effectiveness analyses. The study’s cost-effectiveness analysis is a tool to determine how the cost of RFS impacts the effect, or outcome, of overall cost-savings in patients with indeterminate thyroid nodules. Other limitations include the absence of operative time as a contributor to cost. Since the focus of our study was the cost-effectiveness of intraoperative rapid frozen sections, rather than the cost-effectiveness of varying surgical durations, we did not examine the variation in cost as a function of individual operative time. In addition, we used average operative times at our institution for surgeries of this nature because RFS diagnosis in usually obtained before the surgical wound in closed, thus the addition of intraoperative RFS does not meaningfully add to operative time and time-dependent operative costs. Finally, our study is limited by the decreased use of RFS in the era of lobectomia for papillary thyroid cancer, a topic that is beyond the scope of this paper.

For future studies, additional modalities may replace or supplement rapid frozen sections in guiding operative management of indeterminate thyroid nodules. Molecular markers, gene mutation markers, and gene expression panels have shown to aid in distinguishing benign from malignant thyroid nodules ( 21 ). No patient in our study had records of molecular marker testing of their FNA sample. Identification and correlation of a combination of clinical, sonographic, and cytologic factors may be used to better stratify patients into low and high risk patients ( 5 , 14 , 22 , 23 ). Finally, both ultrasound and pathology are essential for accurate risk stratification and treatment planning in thyroid malignancy. Utilizing high-volume thyroid ultrasonographers and pathologists specializing in thyroid cytopathology and rapid frozen section yields more accurate information for treatment planning.

In conclusion, in the era of outpatient thyroid surgery, we found that routine use of intraoperative rapid frozen section in patients receiving surgery for AUS/FLUS lesions resulted in net savings of $ 80.04 per patient.
